# Differential genome-wide DNA methylation patterns in childhood obesity

**DOI:** 10.1186/s13104-019-4189-0

**Published:** 2019-03-25

**Authors:** Lei Cao-Lei, Guillaume Elgbeili, Moshe Szyf, David P. Laplante, Suzanne King

**Affiliations:** 1 Department of Psychiatry, McGill University and Douglas Hospital Research Centre, Montreal, QC Canada; 2 Douglas Hospital Research Centre, 6875 LaSalle Blvd, Montreal, QC H4H 1R3 Canada; 3 Department of Pharmacology and Therapeutics and Sackler Program for Epigenetics and Developmental Psychobiology, McGill University, Montreal, QC Canada

**Keywords:** Prenatal maternal stress, Genome-wide DNA methylation, Obesity, Project Ice Storm, Immune function

## Abstract

**Objective:**

Exposure to stress during pregnancy may program susceptibility to the development of obesity in offspring. Our goal was to determine whether prenatal maternal stress (PNMS) due to a natural disaster was associated with child obesity, and to compare the DNA methylation profiles in obese versus non-obese children at age 13½ years. Women and their children were involved in the longitudinal natural disaster study—Project Ice Strom, which served as a human model to study PNMS. Blood was collected from 31 children (including five obese children). Infinium HumanMethylation450 BeadChip Array was performed for genome-wide DNA methylation analyses.

**Results:**

Results demonstrated a well-defined obesity-associated genome-wide DNA methylation pattern. There were 277 CpGs, corresponding to 143 genes, were differentially-methylated. IPA analyses revealed 51 canonical pathways, and enrichment of pathways was involved in immune function. Although no significant association was found between PNMS and child obesity, the preliminary data in the study revealed obesity-associated methylation patterns on a genome-wide level in children.

**Electronic supplementary material:**

The online version of this article (10.1186/s13104-019-4189-0) contains supplementary material, which is available to authorized users.

## Introduction

Obesity is a multifactorial condition caused by the complex interaction between genetic and environmental factors. As genetics alone cannot fully explain the rapid global increase in obesity, epigenetic mechanisms have been suggested as playing a role in its etiology [1, 2]. DNA methylation, the best understood epigenetic mechanism, has been suggested to be associated with the development of obesity.

As the intrauterine period is recognized as a critical developmental window, exposure to stress during this period may program susceptibility to the development of obesity. For instance, the Dutch Famine study showed that adverse in utero conditions greatly impacted later adiposity and metabolic risk status [3]. In another study, elevated body mass index (BMI) and percent body fat were observed in young adults whose mothers experienced stressful life events during pregnancy [4]. In addition, maternal bereavement during pregnancy predicted increased rates of offspring obesity [5]. The presumed mechanism may be that prenatal maternal stress (PNMS) induces excessive cortisol which can pass the placental barrier early and could program fetal development of metabolic functioning via the HPA axis [6].

Unlike in animal studies of PNMS, stress in human pregnancy is rarely randomly assigned, which can compromise causal conclusions. Project Ice Storm, a natural disaster study, serves as a quasi-randomized human model to study PNMS [7]. Project Ice Storm was conceived following one of Canada’s worst natural disasters in history: the 1998 Quebec ice storm. Project Ice Storm provides an opportunity to examine the effects of an independent stressor on a number of developmental outcomes prospectively. We have shown that greater objective severity of the women’s exposure to the ice storm was associated with greater BMI in their children at the age of 5½ years, and through the age of 15 [8]. Recently, using selected candidate genes (Type 1 and 2 diabetes-related genes) we reported that DNA methylation mediated the effect of objective severity of exposure to the ice storm on levels of BMI and central adiposity at age 13½ [9]. However, the relationship between PNMS and clinically-defined child obesity at age 13½, and the obesity-related genome-wide DNA methylation profile, has not been explored. The aim of the current study was to determine whether clinically-defined obesity in Project Ice Storm children was associated with PNMS, and to compare obese versus non-obese children at age 13½ years in terms of their DNA methylation on a genome-wide level.

## Main text

### Method

#### Participants

For the assessment of children at age 13½ years in 2011, 36 children agreed to provide a blood sample. DNA samples from two children were not used for DNA methylation analyses due to very low T-cell DNA concentrations. We identified three women who developed gestational diabetes, therefore, their children were excluded in order to avoid confounding effects of maternal gestational diabetes on DNA methylation [10]. A total of 31 children including 5 obese (4 boys and 1 girl) and 26 non-obese (14 boys and 12 girls) were used for further analysis.

#### PNMS

Objective hardship was assessed with the questionnaire, which has been previously published elsewhere [11], by measuring the severity of storm-related events experienced by the pregnant women with four categories of exposure: Threat, Loss, Scope, and Change. Each dimension was scored on a scale of 0–8, ranging from no exposure to high exposure. A total objective hardship score (Storm32) was calculated by summing scores from all four dimensions using McFarlane’s approach [12]. Subjective distress was assessed using a validated French version of the Impact of Event Scale-Revised (IES-R) which has been previously published elsewhere [13]. This 22-item scale, widely used for assessing distress following trauma exposure, describes symptoms from three categories relevant to post-traumatic stress disorder: Intrusive Thoughts, Hyperarousal, and Avoidance. Participants responded on a 5-point Likert scale, from “Not at all” to “Extremely”, the extent to which each behavior described how they felt over the preceding 7 days.

#### Child outcome measures at age 13½ years

During a face-to-face assessment, height, weight and waist measurements were collected following standard guidelines [14], repeating each measure twice and taking the means. Sex- and age-specific BMI (kg/m^2^) percentiles were computed based on World Health Organization growth references adapted for Canadians (http://cpeg-gcep.net/content/who-growth-charts-canada). The children were classified as obese if they exceeded the 97th percentile of BMI.

#### Infinium HumanMethylation450 BeadChip Array

DNA extraction from T-cells was performed using Wizard-Genomic-DNA-Purification-kit (Promega) according to the manufacturer’s instructions. Infinium HumanMethylation450 BeadChip Array and data analysis have been described previously [15].

#### Ingenuity pathway analysis (IPA)

Differentially methylated genes were classified by IPA software. A right-tailed Fisher’s exact test was used to calculate the Gene enrichment. Biological functions with a cut-off p-value < 0.05 were considered statistically significant.

#### Statistical analysis

Infinium HumanMethylation450 BeadChip Array analyses were performed using R packages. All other analyses were performed using SPSS (Version 20, SPSS Inc., Chicago IL, USA). T-test was used to compare the differences between two groups. All p-values reported are two-sided.

### Results

#### Participants’ characteristics

At the time of assessment, these 31 children (18 boys and 13 girls) were on average 13.6 years of age (SD = 0.1), 5 children (16.1%) were classified as obese (4 boys and 1 girl). Their mothers were in their first (n = 9), second (n = 9), or third (n = 6) trimester of pregnancy on January 9, 1998, or conceived within 3 months of the storm (n = 7). The BMI values in the non-obese group ranged from 15.96 to 24.76, while those in the obese group ranged from 26.76 to 36.38. Waist-to-height ratio, skinfolds, BMI and birth weight were significantly greater in children who were classified as obese than in the non-obese group (Table 1).

**Table 1:** Summary of descriptive statistics of study participants characteristics of non-obese and obese samples

	Non-obese sample (N = 26)		Obese sample (N = 5)		t (df); p-value
	Mean	Std. deviation	Mean	Std. deviation	
Children					
Waist-to-height ratio	0.430	0.028	0.551	0.073	t(3.157) = − 3.292; 0.043
Skinfolds-sum	44.029	14.478	85.067	12.615	t(26) = − 5.854; < 0.001
BMI	20.423	2.238	31.449	3.743	t(29) = − 9.030; < 0.001
PNMS					
Objective hardship	11.039	4.219	12.200	4.764	t(29) = − 0.553; 0.584
Subjective distress	9.454	9.639	5.800	3.768	t(29) = 0.826; 0.416
Children’s birth characteristics					
Birth weight (g)	3362.040	714.620	3708.522	105.164	t(27.541) = − 2.303; 0.029
Birth length (cm)	50.716	3.512	50.500	1.581	t(28) = 0.133; 0.895
Ponderal index at birth	25.860	3.483	28.991	2.928	t(28) = − 1.875; 0.071

#### Relationship between PNMS and child obesity

Although the mothers of the obese children tended to have higher objective PNMS than those of non-obese children, these differences were not great enough to be considered statistically significant. Similarly, there were no significant differences in subjective PNMS between the mothers of the obese children and these of the non-obese children (Table 1).

#### Genome-wide DNA methylation pattern in obese group compared to non-obese group

**Fig. 1:**
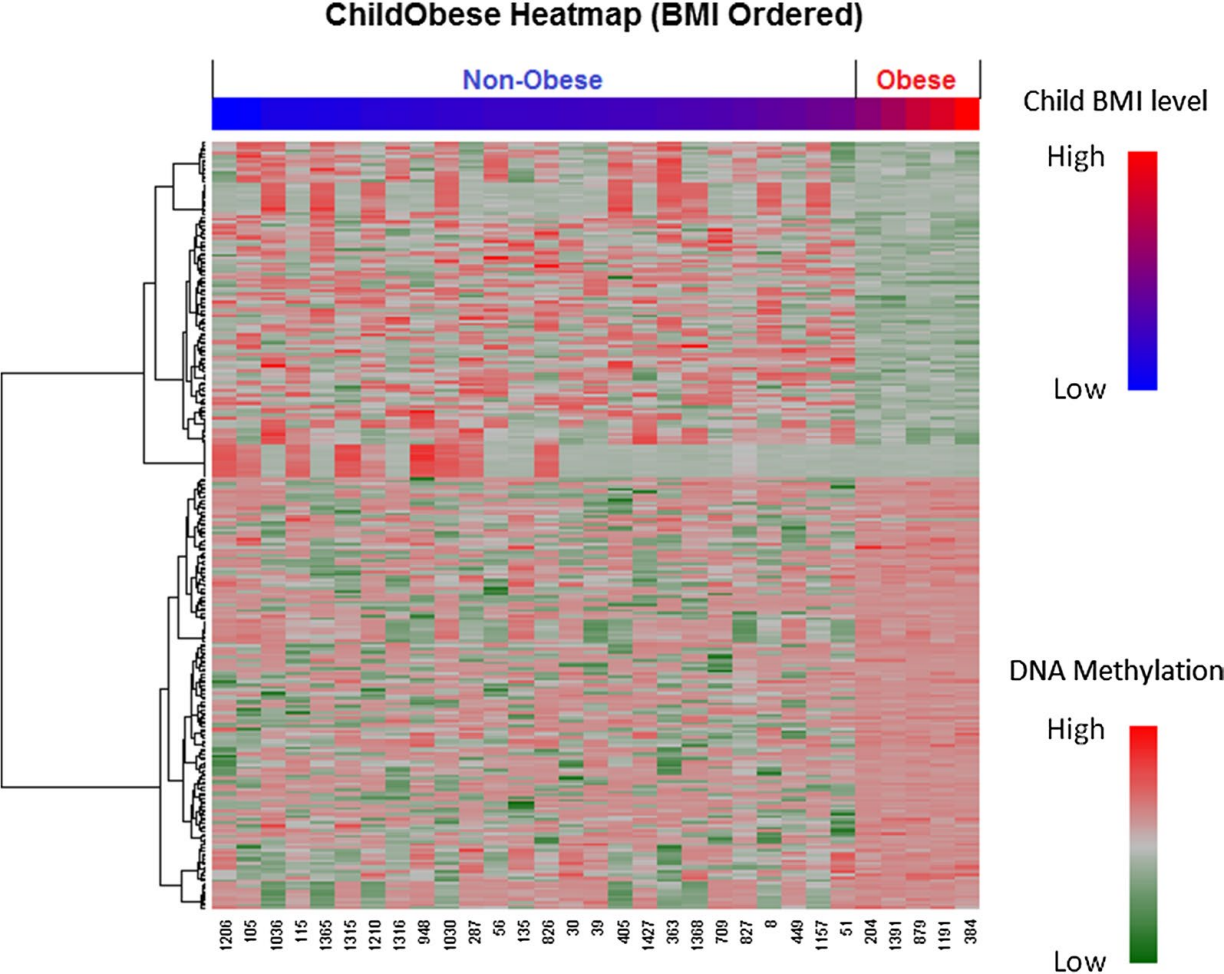
CpG sites which are differentially methylated between obese and non-obese groups. Methylation of the 277 differentially methylated CpGs (p < 0.05, FDR < 0.2) across all 31 individuals are shown in the Heatmap. Each column represents a child and each row a single CpG site. Methylation changes are expressed via a color gradient intensity scale at the lower right-hand corner of the Heatmap: the darkest red indicates the highest DNA methylation level and the darkest green indicates the lowest DNA methylation level. A color gradient intensity scale at the higher right-hand corner expresses children’s BMI level: the darkest red indicates the highest BMI level and the darkest blue indicates the lowest BMI level. The color bar above the Heatmap indicates children categorized by their BMIs: blue indicates a non-obese individual, red indicates an obese individual

In total, significant group differences were observed in 277 CpG sites (p < 0.005, false discovery rate (FDR) < 0.2) (Additional file 1: Table S1) including 10 CpGs with FDR < 0.01 (Table 2) and 4 CpGs with FDR < 0.005. These 277 CpGs were identified to correspond to 143 genes. 122 CpGs were hypo-methylated and 155 CpGs were hyper-methylated in the obese group compared to the non-obese group. Absolute differences (obese group versus non-obese) in DNA methylation beta values for those CpG sites ranged from 0.04 to 0.57. The mean of Cohen’s d effect sizes is 1.028, with minimum and maximum effect sizes of 0.690 and 3.096 respectively. These differentially methylated loci are represented in a Heatmap (Fig. 1). Hierarchical clustering analysis of individual methylation patterns was performed; the results are represented in the dendrogram as shown on the left of the Heatmap. The methylation profiles of the obese and non-obese groups are well distinguished.

**Table 2:** 10 CpG sites (FDR < 0.01) whose DNA methylation levels were significantly different between obese and non-obese group

ID	UCSC_refgene_name	Mean_nonobese	SD non-obese	Mean_obese	SD obese	t	df	p.value	FDR	Mean difference (obese–nonobese)	Cohen’s d
cg16507569	COL11A2	0.840	0.164	0.515	0.012	9.972	26.332	<0.001	<0.001	− 0.324	2.208
cg27289463		0.264	0.090	0.432	0.029	− 7.710	21.432	<0.001	0.001	0.169	2.061
cg25013753	ARHGAP22	0.465	0.318	0.035	0.013	6.877	25.396	<0.001	0.001	− 0.430	1.508
cg19697575		0.596	0.366	0.103	0.065	6.354	28.996	<0.001	0.002	− 0.493	1.494
cg26935333		0.397	0.075	0.293	0.020	6.023	24.897	<0.001	0.007	− 0.104	1.539
cg14007688	DBH	0.577	0.290	0.898	0.011	− 5.620	25.354	<0.001	0.009	0.321	1.232
cg22459517	EPS8L1	0.631	0.194	0.851	0.014	− 5.680	26.244	<0.001	0.009	0.220	1.257
cg25813936	GNE	0.793	0.081	0.886	0.012	− 5.523	28.621	<0.001	0.009	0.093	1.268
cg00320354	TSPAN5	0.758	0.178	0.963	0.027	− 5.538	28.730	<0.001	0.009	0.204	1.276
cg08282428	RBM46	0.680	0.070	0.580	0.022	5.897	21.332	<0.001	0.009	− 0.100	1.578

*COL11A2* collagen type XI alpha 2 chain, *ARHGAP22* rho GTPase activating protein 22, *DBH* dopamine beta-hydroxylase, *EPS8L1* EPS8 like 1, *GNE* glucosamine (UDP-*N*-acetyl)-2-epimerase/*N*-acetylmannosamine kinase, *TSPAN5* tetraspanin 5, *RBM46* RNA binding motif protein 46

#### Differentially methylated pathways related to obesity

Based on the 143 genes differentially methylated between obese and non-obese groups, IPA classified and revealed 52 canonical pathways (Additional file 2: Table S2). The biological functions of these pathways were predominantly involved in immune system. Antigen Presentation Pathway, the primary pathway, included 5 genes: *NLRC5 (NLR Family CARD Domain Containing 5), HLA*-*DRB1 (Major Histocompatibility Complex, Class II, DR Beta 1), HLA*-*B (Major Histocompatibility Complex, Class I, B), TAP1 (Transporter 1, ATP Binding Cassette Subfamily B Member)* and *HLA*-*DRB5 (Major Histocompatibility Complex, Class II, DR Beta 5)* which were found to be associated with obesity in the current study.

### Discussion

DNA methylation, the best understood epigenetic mechanism, has been suggested to be associated with the development of obesity. As reviewed by Demetriou et al. [16], studies on obesity in childhood or adolescence demonstrate significant associations between childhood or adolescent BMI/obesity and DNA methylation in peripheral blood.

Our results demonstrated a well-defined obesity-associated genome-wide DNA methylation pattern. There were 277 CpGs, corresponding to 143 genes that were differentially-methylated. IPA analyses revealed 51 canonical pathways and enrichment of pathways was involved in immune function.

The top CpG cg16507569 with FDR < 0.001 is located on the collagen type XI alpha 2 (COL11A2) gene which encodes one of the two alpha chains of type XI collagen. This gene has been reported to be involved in metabolic syndrome. For instance, in a study investigating the contribution of genetic and epigenetic factors in the development of the metabolic syndrome, 2 methylation quantitative trait loci (meQTL) disrupting CpG sites located within the COL11A2 gene were revealed for associations with the metabolic syndrome and its components in obese individuals, and interestingly, CpG cg16507569 showed a reduction in methylation levels in the presence of a minor allele of rs114894582 [17]. The CpG cg25013753 is located on Rho GTPase Activating Protein 22 (ARHGAP22) gene which has been identified as one of the T2D risk loci [18, 19] and also implicated in a novel insulin regulated pathway [20, 21]. The CpG cg14007688 is located on dopamine beta-hydroxylase (DBH) gene. Mice with inactivation of the dopamine beta-hydroxylase gene (Dbh-null mice) gain weight on a high fat diet comparing to the control mice [22]. Similarly, the mouse model overexpressing NPY driven by DBH gene promoter (OE-NPY^DBH^) displays obesity and impaired glucose metabolism [23–25].

Among the 143 differentially methylated genes in this study, the *HLA* set is of particular interest. Evidence is growing that the *HLA* set is associated with obesity. For example, the *HLA*-*DQ* genotype was reported to increase risk for obesity among 2–4 year-old children with genetic risk for type-1 diabetes [26]. Similarly, the *HLA*-*DQ* genotype was found to be associated with increased BMI in type-1 diabetes children [27]. Furthermore, the *HLA* genotype was observed to interact with BMI status in relation to the risk of developing multiple sclerosis [28].

Moreover, we identified 52 canonical obesity-associated pathways from the 143 genes that were differentially methylated between the obese and non-obese groups. The top pathway was the Antigen Presentation Pathway. It is well known that the major histocompatibility (MHC) class I Antigen Presentation Pathway plays an important role in altering the immune system in response to virally infected cells. Furthermore, this pathway has been reported to play an essential role in obesity-induced adipose inflammation [29]. Similarly, this pathway was observed in visceral adipose tissue in obese men discordant for metabolic disturbances [30]. Interestingly, by comparing our finding with the latter study, we observed 7 common pathways including Antigen Presentation Pathway, Graft-versus-Host Disease Signaling, Autoimmune Thyroid Disease Signaling, Cdc42 Signaling, Dendritic Cell Maturation, Calcium-induced T Lymphocyte Apoptosis, and Signaling by Rho Family GTPases. The considerable overlap at the pathway level between our finding from T-cells and that from adipose tissue suggests that peripheral blood tissue holds great promise for the further application of DNA methylation signatures of psychosocial exposures for determining biomarkers of methylation status.

It is important to note pathways involved in immune system in the current study are prominent. Impaired immune functions have been described in both humans and genetically obese rodents. Obesity has been associated with inflammation, and adipokines modulate immune function [31]. Therefore, our finding that immune functions were involved in obesity-associated pathways is in accordance with that reported in our recent immunity exploration in which we have shown that objective PNMS significantly predicted reductions of CD4+ lymphocyte and increases in TNF-α, IL-1β, and IL-6 levels, and an enhancement of the Th2 cytokines IL-4 and IL-13 in the Project Ice Storm cohort [32] using the same blood draws as for the epigenetic study. Therefore, our finding presents evidence to support the crucial and widely role of immune system on obesity.

## Limitations

(1) The small sample size of only 5 obese children is major issue that limits the significance of the findings; furthermore, the sex specificity of the DNA methylation cannot be tested based on such cohort; (2) We have no access to detect gene expression of relevant genes due to the lack of RNA samples; (3) Although the current study revealed obesity-associated methylation differences on a genome-wide level, we cannot draw any conclusions about whether the methylation differences are the cause, or the consequence, of obesity; (4) Because the DNA analyzed was obtained from the children at age 13½, we cannot rule out the possibility that confounders, such as prenatal maternal anxiety, which differed significantly between the groups, could be responsible for our results on both obesity patterns and DNA methylation levels. In summary, future studies with larger sample sizes are warranted to address these issues, and further investigation is required to better test the causality between DNA methylation and obesity.

## Additional files

**Additional file 1: Table S1.** 277 CpG sites (FDR < 0.2) whose DNA methylation levels were significantly different between obese and non-obese group. Significant group differences were observed in 277 CpG sites (p < 0.005, FDR < 0.2) including 10 CpGs with FDR < 0.01 and 4 CpGs with FDR < 0.005. These 277 CpGs were identified to correspond to 143 genes.

**Additional file 2: Table S2.** 52 canonical pathways classified with IPA. IPA classified and revealed 52 canonical pathways based on the 143 genes differentially methylated between obese and non-obese groups. The biological functions of these pathways were predominantly involved in immune system.

## Abbreviation

PNMS: Prenatal maternal stress
IPA: Ingenuity pathway analysis
BMI: Body mass index
FDR: False discovery rate
