# Supplementary material for: Differential genome-wide DNA methylation patterns in childhood obesity
Source: BMC Res Notes. 2019 Mar 25;12:174. doi: 10.1186/s13104-019-4189-0 (PMC6434834; doi:10.1186/s13104-019-4189-0)
Supplement: Supplementary file 1 — Additional file 1: Table S1. 277 CpG sites (FDR < 0.2) whose DNA methylation levels were significantly different between obese and non-obese group. Significant group differences were observed in 277 CpG sites (p < 0.005, FDR < 0.2) including 10 CpGs with FDR < 0.01 and 4 CpGs with FDR < 0.005. These 277 CpGs were identified to correspond to 143 genes. [file 13104_2019_4189_MOESM1_ESM.pdf]

Table S1: 277 CpG sites (FDR<0.2) whose DNA methylation levels were significantly different between obese and non-obese group.

ID	UCSC_REFGENE_NAME	Mean_NonObese	SD Non-Obese	Mean_Obese	SD Obese	t	df	p.value	FDR	Mean difference (obese-nonobese)	Cohen's d
cg16507569	COL11A2	0.840	0.164	0.515	0.012	9.972	26.332	<0.001	<0.001	-0.324	2.208
cg27289463		0.264	0.090	0.432	0.029	-7.710	21.432	<0.001	0.001	0.169	2.061
cg25013753	ARHGAP22	0.465	0.318	0.035	0.013	6.877	25.396	<0.001	0.001	-0.430	1.508
cg19697575		0.596	0.366	0.103	0.065	6.354	28.996	<0.001	0.002	-0.493	1.494
cg26935333		0.397	0.075	0.293	0.020	6.023	24.897	<0.001	0.007	-0.104	1.539
cg14007688	DBH	0.577	0.290	0.898	0.011	-5.620	25.354	<0.001	0.009	0.321	1.232
cg22459517	EPS8L1	0.631	0.194	0.851	0.014	-5.680	26.244	<0.001	0.009	0.220	1.257
cg25813936	GNE	0.793	0.081	0.886	0.012	-5.523	28.621	<0.001	0.009	0.093	1.268
cg00320354	TSPAN5	0.758	0.178	0.963	0.027	-5.538	28.730	<0.001	0.009	0.204	1.276
cg08282428	RBM46	0.680	0.070	0.580	0.022	5.897	21.332	<0.001	0.009	-0.100	1.578
cg10977910	TTLL7	0.387	0.137	0.143	0.057	6.584	14.768	<0.001	0.011	-0.244	1.960
cg27413643	ANKRD27;RGS9BP	0.301	0.195	0.092	0.014	5.381	26.266	<0.001	0.012	-0.209	1.191
cg06549275	ASAP2	0.638	0.067	0.723	0.019	-5.441	24.041	<0.001	0.012	0.085	1.406
cg26658743		0.747	0.060	0.665	0.020	5.651	20.755	<0.001	0.012	-0.083	1.524
cg05248234	FSCN2	0.505	0.372	0.114	0.037	5.228	27.163	<0.001	0.013	-0.391	1.169
cg00340052	AGAP1	0.819	0.052	0.719	0.024	6.692	12.601	<0.001	0.013	-0.100	2.099
cg19181528		0.732	0.142	0.891	0.032	-5.066	27.809	<0.001	0.017	0.159	1.239
cg01835922		0.354	0.309	0.042	0.004	5.144	25.035	<0.001	0.018	-0.312	1.124
cg03877767	GREB1	0.712	0.072	0.615	0.024	5.374	19.827	<0.001	0.020	-0.096	1.468
cg07248242	TSPAN9	0.608	0.085	0.522	0.014	4.825	28.978	<0.001	0.024	-0.086	1.126
cg00152799	TXNL1	0.353	0.065	0.251	0.027	5.749	14.814	<0.001	0.024	-0.102	1.710
cg06916011	EPS8L1	0.297	0.062	0.361	0.012	-4.803	28.884	<0.001	0.025	0.064	1.141

cg21158163		0.759	0.144	0.909	0.029	-4.799	28.690	<0.001	0.025	0.150	1.149
cg21040096	RPH3AL	0.781	0.150	0.931	0.027	-4.715	28.985	<0.001	0.027	0.150	1.111
cg09949906	HLA-DRB1	0.598	0.333	0.937	0.065	-4.732	28.798	<0.001	0.027	0.338	1.129
cg14377951	GSTM5	0.335	0.090	0.438	0.026	-4.890	23.595	<0.001	0.027	0.103	1.271
cg01520956	FAM47E	0.451	0.058	0.519	0.017	-4.902	22.919	<0.001	0.028	0.068	1.285
cg23336892		0.421	0.214	0.214	0.032	4.662	28.697	<0.001	0.028	-0.207	1.073
cg05808124		0.824	0.076	0.897	0.011	-4.660	28.743	<0.001	0.028	0.074	1.074
cg10764750		0.630	0.115	0.771	0.038	-4.952	20.259	<0.001	0.031	0.141	1.345
cg06508867		0.304	0.073	0.227	0.018	4.650	26.485	<0.001	0.033	-0.076	1.162
cg26590106	HLA-DRB1	0.574	0.347	0.240	0.063	4.522	28.978	<0.001	0.036	-0.334	1.067
cg27112983		0.755	0.167	0.926	0.040	-4.574	27.036	<0.001	0.036	0.171	1.134
cg20756026	HEXDC	0.618	0.244	0.839	0.011	-4.591	25.537	<0.001	0.036	0.221	1.008
cg07618759	COL4A2	0.577	0.312	0.859	0.023	-4.554	26.327	<0.001	0.036	0.282	1.008
cg06052372		0.649	0.302	0.923	0.011	-4.608	25.362	<0.001	0.036	0.274	1.010
cg11317459		0.106	0.107	0.010	0.009	4.521	26.654	<0.001	0.037	-0.096	1.005
cg11251367	FMN2	0.327	0.334	0.029	0.009	4.531	25.179	<0.001	0.038	-0.298	0.991
cg07437923	DNAJA3	0.319	0.325	0.029	0.014	4.528	25.459	<0.001	0.038	-0.290	0.993
cg05834845	MUC4	0.702	0.193	0.885	0.038	-4.418	28.821	<0.001	0.039	0.183	1.053
cg27362989	HLA-DRB5	0.527	0.298	0.104	0.128	5.179	14.218	<0.001	0.039	-0.423	1.560
cg23477460		0.168	0.062	0.108	0.014	4.367	27.908	<0.001	0.045	-0.060	1.066
cg05545777		0.424	0.074	0.502	0.022	-4.493	23.204	<0.001	0.046	0.078	1.173
cg15586842	ANXA8L2	0.796	0.109	0.923	0.038	-4.642	19.234	<0.001	0.048	0.127	1.279
cg12112110	TNXB;TNXB	0.590	0.066	0.505	0.026	4.832	15.803	<0.001	0.051	-0.085	1.409
cg13752114	MUC4	0.833	0.146	0.960	0.016	-4.281	27.429	<0.001	0.054	0.126	0.961
cg04417708	ZZEF1	0.758	0.200	0.928	0.010	-4.318	25.632	<0.001	0.054	0.170	0.949

cg05276469		0.301	0.180	0.146	0.021	4.245	27.864	<0.001	0.055	-0.155	0.959
cg23153338	OR52I1	0.610	0.058	0.669	0.017	-4.357	23.260	<0.001	0.056	0.059	1.137
cg12077433	SCHIP1	0.660	0.264	0.884	0.020	-4.250	26.313	<0.001	0.057	0.223	0.941
cg17876294		0.539	0.157	0.406	0.012	4.249	26.443	<0.001	0.057	-0.133	0.942
cg01943931		0.581	0.284	0.824	0.033	-4.209	27.755	<0.001	0.057	0.243	0.950
cg07099606		0.850	0.124	0.953	0.007	-4.215	25.848	<0.001	0.057	0.103	0.928
cg05130642	EPPK1	0.547	0.371	0.857	0.024	-4.226	25.998	<0.001	0.057	0.311	0.932
cg02132667		0.474	0.072	0.413	0.009	4.164	27.919	<0.001	0.057	-0.061	0.942
cg15602423	HLA-DRB1	0.552	0.328	0.835	0.052	-4.143	28.900	<0.001	0.057	0.283	0.961
cg12733396	COL9A2	0.788	0.083	0.860	0.014	-4.138	28.978	<0.001	0.057	0.072	0.966
cg04422742	HLA-DRB6	0.528	0.250	0.307	0.046	4.149	28.970	<0.001	0.057	-0.221	0.979
cg10434728	IQGAP1	0.686	0.062	0.630	0.013	4.175	28.420	<0.001	0.057	-0.056	1.008
cg24629711	MUSTN1	0.736	0.210	0.910	0.014	-4.179	26.136	<0.001	0.058	0.174	0.923
cg10204320	MTMR9L	0.506	0.066	0.564	0.013	-4.122	28.850	<0.001	0.058	0.058	0.981
cg04222842	SRP9	0.150	0.057	0.099	0.013	4.130	27.952	<0.001	0.059	-0.052	1.007
cg11404906	HLA-DRB1	0.632	0.346	0.934	0.064	-4.100	28.969	<0.001	0.059	0.302	0.968
cg18111114	HLA-DRB5	0.165	0.192	0.007	0.010	4.162	25.668	<0.001	0.060	-0.158	0.915
cg21792493		0.484	0.273	0.263	0.010	4.112	25.359	<0.001	0.061	-0.221	0.901
cg00861214	CR1L	0.596	0.262	0.809	0.014	-4.116	25.695	<0.001	0.061	0.213	0.905
cg10306192	MMP27	0.272	0.321	0.010	0.009	4.138	25.213	<0.001	0.061	-0.261	0.906
cg18578876	GPR109B	0.768	0.134	0.878	0.014	-4.078	27.283	<0.001	0.061	0.110	0.914
cg01736133		0.660	0.086	0.732	0.012	-4.080	28.551	<0.001	0.061	0.073	0.935
cg18918831	MUC4	0.683	0.165	0.842	0.046	-4.136	24.331	<0.001	0.061	0.159	1.065
cg22984586	CCR5	0.350	0.199	0.551	0.062	-4.205	21.975	<0.001	0.061	0.201	1.116
cg17715141		0.623	0.077	0.541	0.026	4.289	20.044	<0.001	0.061	-0.082	1.168
cg07839457	NLRC5	0.434	0.073	0.353	0.026	4.396	18.065	<0.001	0.061	-0.081	1.233

cg06711418	MT2A	0.287	0.178	0.142	0.017	4.046	26.958	<0.001	0.065	-0.145	0.903
cg07741840	KDM2B	0.608	0.290	0.841	0.016	-4.054	25.763	<0.001	0.066	0.232	0.892
cg19491478		0.666	0.072	0.749	0.028	-4.402	16.665	<0.001	0.066	0.082	1.264
cg13905298		0.756	0.227	0.937	0.008	-4.048	25.346	<0.001	0.068	0.181	0.887
cg19353052	DTX1	0.696	0.257	0.901	0.014	-4.033	25.749	<0.001	0.068	0.205	0.887
cg11466708	C22orf43	0.685	0.211	0.855	0.022	-3.995	27.319	<0.001	0.068	0.170	0.895
cg24340935		0.369	0.058	0.304	0.022	4.346	16.982	<0.001	0.068	-0.065	1.241
cg10632894	HLA-DRB1	0.744	0.256	0.946	0.013	-4.005	25.608	<0.001	0.071	0.203	0.880
cg25550823		0.753	0.235	0.938	0.006	-4.003	25.155	<0.001	0.072	0.185	0.876
cg01414268		0.259	0.134	0.141	0.031	3.957	27.473	<0.001	0.072	-0.118	0.974
cg22627029	HLA-DRB6	0.304	0.333	0.042	0.020	3.965	25.892	0.001	0.073	-0.261	0.874
cg13910785	HLA-DRB1	0.350	0.422	0.018	0.020	3.984	25.576	<0.001	0.073	-0.332	0.875
cg17745575	FAM106A	0.608	0.233	0.793	0.027	-3.903	27.764	0.001	0.073	0.184	0.881
cg24233293		0.672	0.123	0.770	0.015	-3.910	27.991	0.001	0.073	0.098	0.886
cg05653481	LOC645332	0.740	0.120	0.836	0.016	-3.917	28.259	0.001	0.073	0.096	0.892
cg13522370		0.514	0.322	0.772	0.048	-3.879	28.716	0.001	0.073	0.259	0.893
cg12890750	VTRNA1-2	0.474	0.164	0.606	0.025	-3.895	28.777	0.001	0.073	0.132	0.899
cg15251140		0.586	0.332	0.855	0.049	-3.905	28.698	0.001	0.073	0.269	0.899
cg01310397	MUC4	0.800	0.097	0.879	0.016	-3.886	28.914	0.001	0.073	0.079	0.902
cg19218082	COL4A2	0.673	0.280	0.902	0.049	-3.870	29.000	0.001	0.073	0.229	0.908
cg17479576	FAM160A1	0.829	0.138	0.956	0.037	-3.980	25.103	0.001	0.073	0.127	1.014
cg12252547	MAL2	0.426	0.181	0.593	0.051	-3.969	24.073	0.001	0.073	0.167	1.025
cg14785464	HLCS	0.490	0.087	0.374	0.041	4.623	12.269	0.001	0.073	-0.116	1.463
cg07415359	LDHC	0.819	0.071	0.875	0.009	-3.857	28.272	0.001	0.076	0.056	0.878

cg26036029	HLA-DRB1	0.848	0.101	0.929	0.016	-3.851	28.898	0.001	0.076	0.081	0.893
cg09399716		0.610	0.167	0.748	0.033	-3.846	28.797	0.001	0.076	0.138	0.917
cg23490161	SDHA;CCDC127	0.126	0.150	0.011	0.002	3.905	25.069	0.001	0.076	-0.115	0.853
cg12109260		0.901	0.058	0.781	0.041	5.578	7.579	0.001	0.076	-0.120	2.208
cg08950364		0.643	0.216	0.810	0.022	-3.856	27.192	0.001	0.076	0.168	0.863
cg13275603	ENC1	0.276	0.080	0.358	0.028	-4.061	18.738	0.001	0.080	0.082	1.127
cg13176198		0.851	0.079	0.749	0.037	4.506	12.295	0.001	0.080	-0.102	1.425
cg13554818	PHLDB3	0.602	0.081	0.674	0.021	-3.856	25.615	0.001	0.080	0.072	0.976
cg27057480	ARHGAP22	0.343	0.212	0.148	0.062	3.907	23.264	0.001	0.080	-0.196	1.020
cg05308244	RIMBP2	0.643	0.321	0.886	0.017	-3.839	25.714	0.001	0.081	0.243	0.844
cg13017022	ZMAT2	0.709	0.301	0.941	0.034	-3.806	27.666	0.001	0.081	0.232	0.857
cg16095660	PGAP2	0.840	0.083	0.908	0.017	-3.792	28.555	0.001	0.081	0.068	0.912
cg24960960	SDHAP3	0.121	0.149	0.008	0.006	3.839	25.376	0.001	0.081	-0.113	0.841
cg25210835	GSTM5	0.300	0.160	0.482	0.066	-4.227	15.026	0.001	0.081	0.182	1.251
cg07582993	UBOX5;FASTKD5	0.524	0.082	0.448	0.024	3.892	22.859	0.001	0.081	-0.076	1.021
cg22999099	BOK	0.906	0.100	0.984	0.014	-3.763	28.606	0.001	0.083	0.078	0.863
cg06627617	ASAP2	0.498	0.116	0.631	0.049	-4.228	14.649	0.001	0.083	0.133	1.262
cg04335714		0.471	0.065	0.523	0.012	-3.748	28.937	0.001	0.084	0.052	0.887
cg27378537	SDHAP3	0.152	0.120	0.062	0.005	3.802	25.500	0.001	0.084	-0.090	0.835
cg14398295		0.658	0.089	0.560	0.036	4.129	15.712	0.001	0.085	-0.098	1.206
cg21167402	SDHAP3	0.170	0.165	0.044	0.021	3.746	28.095	0.001	0.085	-0.126	0.850
cg14317230	MTDH	0.932	0.056	0.991	0.021	-4.037	17.201	0.001	0.085	0.059	1.148
cg09858108		0.437	0.106	0.294	0.054	4.526	11.151	0.001	0.085	-0.144	1.484
cg08422420	SDHAP3	0.133	0.170	0.006	0.003	3.781	25.056	0.001	0.086	-0.126	0.826
cg15048554	BTBD11	0.225	0.256	0.035	0.017	3.763	26.080	0.001	0.086	-0.191	0.831
cg10395519	MTHFD1L	0.830	0.059	0.889	0.021	-3.962	18.637	0.001	0.086	0.059	1.101

cg21931717	SDHAP3	0.089	0.118	0.001	0.002	3.773	25.106	0.001	0.086	-0.087	0.825
cg24875593	PDXK	0.891	0.094	0.962	0.010	-3.732	27.510	0.001	0.086	0.071	0.839
cg17369694	HLA-DRB5	0.304	0.363	0.037	0.019	3.739	25.672	0.001	0.086	-0.268	0.822
cg08578320	HLA-DRB1	0.680	0.297	0.902	0.030	-3.717	27.216	0.001	0.086	0.222	0.832
cg06488536		0.530	0.063	0.482	0.011	3.689	28.999	0.001	0.086	-0.049	0.865
cg06085042	MIR570	0.719	0.168	0.854	0.035	-3.705	28.543	0.001	0.086	0.135	0.891
cg07341007	MUC4	0.699	0.126	0.803	0.029	-3.706	27.670	0.001	0.086	0.104	0.909
cg11274172	UVRAG	0.620	0.065	0.677	0.018	-3.776	24.298	0.001	0.086	0.057	0.972
cg09476440	KIAA1324	0.796	0.139	0.942	0.054	-4.023	16.557	0.001	0.086	0.146	1.157
cg26839410	NTM	0.792	0.187	0.930	0.014	-3.720	26.313	0.001	0.087	0.138	0.824
cg26472636	SDHAP3	0.198	0.169	0.072	0.017	3.705	27.269	0.001	0.087	-0.126	0.830
cg08778598	SDHAP3	0.158	0.200	0.013	0.005	3.701	25.193	0.001	0.091	-0.145	0.810
cg15706692		0.170	0.198	0.026	0.006	3.701	25.218	0.001	0.091	-0.144	0.810
cg02305164	ATP8B3	0.756	0.232	0.925	0.008	-3.711	25.301	0.001	0.091	0.169	0.813
cg03398919		0.655	0.263	0.847	0.016	-3.690	25.911	0.001	0.091	0.192	0.813
cg07093060		0.740	0.212	0.577	0.036	3.651	28.970	0.001	0.091	-0.163	0.851
cg01099512	CTSG	0.254	0.059	0.301	0.012	-3.650	28.644	0.001	0.091	0.047	0.875
cg17646820		0.248	0.062	0.194	0.018	3.736	23.869	0.001	0.091	-0.054	0.967
cg21805179	MPHOSPH9	0.254	0.088	0.176	0.026	3.754	22.919	0.001	0.091	-0.078	0.984
cg19466822	BCL11B	0.539	0.105	0.447	0.031	3.725	23.425	0.001	0.093	-0.092	0.970
cg06721546	SDHAP3	0.142	0.111	0.061	0.007	3.672	25.921	0.001	0.093	-0.081	0.810
cg25140213	HLA-DRB6	0.442	0.313	0.206	0.048	3.626	28.797	0.001	0.093	-0.236	0.837
cg23914255	AACS	0.750	0.210	0.906	0.027	-3.619	28.229	0.001	0.094	0.155	0.823
cg19774683	HLA-DRB6	0.357	0.419	0.046	0.055	3.624	28.244	0.001	0.094	-0.311	0.825
cg23643375	SP6	0.734	0.158	0.860	0.034	-3.624	28.172	0.001	0.094	0.126	0.880
cg26299084	PRDM8	0.324	0.081	0.230	0.036	4.115	13.444	0.001	0.094	-0.093	1.262
cg00819048		0.762	0.220	0.920	0.011	-3.652	25.618	0.001	0.095	0.159	0.802

cg02423105	CDH26	0.189	0.080	0.102	0.034	3.991	14.705	0.001	0.099	-0.087	1.190
cg01010328	TTC24	0.310	0.069	0.388	0.030	-4.043	13.955	0.001	0.099	0.077	1.225
cg27283514		0.748	0.056	0.659	0.035	4.688	8.731	0.001	0.099	-0.089	1.713
cg07100532	ENOSF1	0.853	0.089	0.715	0.054	4.651	8.844	0.001	0.100	-0.139	1.689
cg04996388	MYADML2	0.880	0.117	0.965	0.012	-3.582	27.404	0.001	0.102	0.085	0.804
cg23667563		0.799	0.195	0.942	0.026	-3.570	28.326	0.001	0.102	0.143	0.814
cg27149073	SDHAP3	0.191	0.164	0.074	0.010	3.599	25.941	0.001	0.102	-0.117	0.793
cg16854281		0.615	0.158	0.735	0.028	-3.554	28.993	0.001	0.102	0.119	0.836
cg05531409	CPNE4	0.713	0.249	0.890	0.016	-3.581	25.995	0.001	0.106	0.177	0.790
cg03987818		0.747	0.265	0.935	0.017	-3.571	26.048	0.001	0.108	0.188	0.788
cg17626301	TAP1	0.418	0.094	0.337	0.028	3.638	22.469	0.001	0.108	-0.081	0.959
cg12434901	KCNH6	0.632	0.323	0.860	0.018	-3.559	25.788	0.001	0.111	0.227	0.783
cg09139047	HLA-DRB1	0.683	0.349	0.930	0.026	-3.552	26.316	0.001	0.111	0.247	0.786
cg19961153	ALPPL2	0.437	0.301	0.648	0.010	-3.563	25.307	0.001	0.111	0.211	0.780
cg01341801	HLA-DRB5	0.301	0.375	0.038	0.024	3.538	26.005	0.002	0.114	-0.263	0.781
cg14210114	IGSF8	0.875	0.099	0.945	0.007	-3.507	26.356	0.002	0.120	0.069	0.777
cg05237260		0.370	0.103	0.482	0.045	-3.908	13.672	0.002	0.120	0.112	1.192
cg19409579	PRDM8	0.341	0.103	0.195	0.060	4.366	9.384	0.002	0.120	-0.146	1.540
cg12639429	TTC39C	0.581	0.070	0.514	0.026	3.706	17.527	0.002	0.121	-0.067	1.048
cg24913825		0.452	0.080	0.371	0.033	3.800	15.392	0.002	0.121	-0.082	1.117
cg20052865	SNORD114-1	0.700	0.052	0.631	0.028	4.230	10.132	0.002	0.122	-0.069	1.442
cg10187601	ST7	0.805	0.143	0.913	0.031	-3.460	28.070	0.002	0.124	0.108	0.842
cg16034541	MUC4	0.634	0.197	0.789	0.050	-3.482	26.330	0.002	0.124	0.155	0.872
cg16819848	ZFYVE28	0.451	0.259	0.231	0.080	3.549	22.084	0.002	0.126	-0.220	0.940
cg01033205		0.808	0.186	0.935	0.012	-3.465	26.027	0.002	0.130	0.128	0.765
cg13202221	TM2D3	0.467	0.052	0.403	0.027	4.068	10.972	0.002	0.130	-0.064	1.342

cg09858862	ID3	0.751	0.083	0.811	0.014	-3.420	28.983	0.002	0.130	0.060	0.799
cg14408831		0.713	0.297	0.915	0.010	-3.462	25.309	0.002	0.131	0.202	0.758
cg08914678	PDIA2	0.596	0.258	0.774	0.026	-3.434	27.302	0.002	0.131	0.178	0.770
cg00138041	PRDM8	0.698	0.080	0.587	0.047	4.281	9.277	0.002	0.131	-0.112	1.519
cg01882774	OTUB2	0.797	0.134	0.891	0.018	-3.417	28.300	0.002	0.131	0.094	0.778
cg22961278	PRDM8	0.366	0.089	0.276	0.037	3.746	14.984	0.002	0.131	-0.090	1.110
cg01124477		0.757	0.060	0.689	0.029	3.937	12.106	0.002	0.131	-0.068	1.252
cg08241318		0.602	0.059	0.647	0.014	-3.415	27.501	0.002	0.133	0.045	0.840
cg03605463		0.884	0.094	0.968	0.032	-3.559	19.574	0.002	0.134	0.083	0.976
cg03372334	MBNL2	0.692	0.324	0.913	0.027	-3.418	26.607	0.002	0.134	0.221	0.759
cg26969150	POTEA	0.777	0.153	0.881	0.013	-3.418	26.716	0.002	0.134	0.104	0.760
cg02276823	MMD2	0.507	0.087	0.435	0.026	3.476	22.549	0.002	0.136	-0.072	0.915
cg18713687	MUC4	0.846	0.083	0.912	0.022	-3.426	25.055	0.002	0.137	0.066	0.873
cg23489137	RBMS1	0.699	0.114	0.600	0.038	3.524	20.258	0.002	0.137	-0.099	0.957
cg14199961		0.289	0.057	0.170	0.048	4.938	6.450	0.002	0.137	-0.119	2.195
cg20741528	CCDC127;SDHA	0.103	0.076	0.052	0.004	3.411	25.597	0.002	0.137	-0.051	0.749
cg03224005		0.706	0.273	0.890	0.017	-3.403	25.949	0.002	0.137	0.184	0.750
cg13633881	ADAMTS13	0.768	0.183	0.894	0.021	-3.386	27.660	0.002	0.137	0.126	0.763
cg01517384	HLA-B	0.394	0.070	0.314	0.034	3.918	11.626	0.002	0.137	-0.081	1.265
cg12420107	VTRNA1-1	0.344	0.123	0.432	0.023	-3.358	28.950	0.002	0.139	0.088	0.794
cg03721887	CTDP1	0.668	0.361	0.908	0.010	-3.397	25.217	0.002	0.139	0.241	0.743
cg00247094	TBC1D16	0.745	0.214	0.890	0.020	-3.376	26.990	0.002	0.139	0.145	0.754
cg03226114		0.512	0.077	0.439	0.030	3.615	16.316	0.002	0.139	-0.073	1.044
cg02157827	FLOT1;IER3	0.212	0.103	0.324	0.047	-3.791	12.977	0.002	0.139	0.111	1.177
cg05865327	PPP2R5C	0.763	0.189	0.889	0.014	-3.369	26.263	0.002	0.140	0.126	0.745

cg04173211	IL16	0.871	0.123	0.953	0.011	-3.359	26.753	0.002	0.140	0.083	0.747
cg08912652	SNX19	0.831	0.211	0.973	0.018	-3.366	26.689	0.002	0.140	0.142	0.749
cg24450063	SLC25A44	0.250	0.121	0.334	0.017	-3.341	28.614	0.002	0.140	0.083	0.767
cg19596870	SERPINB6	0.661	0.298	0.868	0.047	-3.334	28.865	0.002	0.140	0.207	0.772
cg12333160		0.148	0.096	0.079	0.020	3.341	28.343	0.002	0.140	-0.070	0.808
cg01446217	ERMP1	0.287	0.066	0.346	0.024	-3.538	18.146	0.002	0.140	0.059	0.991
cg04227007	LOC284412	0.749	0.195	0.884	0.029	-3.332	28.688	0.002	0.141	0.135	0.766
cg09834503		0.531	0.069	0.474	0.022	3.445	21.190	0.002	0.141	-0.058	0.924
cg19290738	AP3D1	0.346	0.087	0.248	0.042	3.832	11.959	0.002	0.141	-0.098	1.224
cg08474748		0.499	0.082	0.403	0.042	3.891	11.168	0.002	0.142	-0.096	1.275
cg11861387	PPYR1	0.268	0.234	0.112	0.019	3.345	26.510	0.002	0.143	-0.156	0.742
cg24824686	PTPRE	0.713	0.066	0.766	0.020	-3.402	22.675	0.002	0.143	0.053	0.894
cg09885502	GNAS	0.553	0.292	0.127	0.185	4.242	8.434	0.003	0.144	-0.426	1.579
cg04995300		0.248	0.068	0.196	0.018	3.351	24.978	0.003	0.147	-0.052	0.855
cg10983013		0.778	0.155	0.889	0.032	-3.302	28.490	0.003	0.147	0.111	0.796
cg14293674		0.769	0.215	0.911	0.012	-3.330	25.775	0.003	0.149	0.142	0.733
cg22676075		0.579	0.123	0.498	0.011	3.298	26.937	0.003	0.154	-0.081	0.736
cg25010115	MYADML2	0.896	0.080	0.950	0.012	-3.275	28.649	0.003	0.154	0.054	0.753
cg05524354	PTPRE	0.641	0.084	0.714	0.030	-3.443	18.899	0.003	0.154	0.073	0.953
cg03847896		0.585	0.149	0.771	0.082	-3.950	9.964	0.003	0.154	0.185	1.356
cg01760090		0.574	0.101	0.465	0.048	3.731	12.211	0.003	0.154	-0.109	1.183
cg10648815	LAIR2	0.564	0.079	0.629	0.025	-3.363	21.878	0.003	0.156	0.064	0.894
cg11881038	OPRM1	0.712	0.229	0.861	0.012	-3.300	25.644	0.003	0.156	0.149	0.725
cg17763566	HLA-DPB2	0.765	0.184	0.885	0.011	-3.295	25.906	0.003	0.156	0.120	0.726
cg10601372	WDR18	0.903	0.132	0.989	0.009	-3.287	26.074	0.003	0.156	0.086	0.726

cg13318793	HLA-DRB5	0.875	0.095	0.947	0.026	-3.305	24.844	0.003	0.156	0.073	0.845
cg04235768	PRDM8	0.238	0.065	0.143	0.042	4.206	8.117	0.003	0.156	-0.096	1.599
cg08861434		0.772	0.222	0.923	0.034	-3.253	28.818	0.003	0.156	0.150	0.752
cg03623097	ARMS2	0.548	0.081	0.641	0.042	-3.799	11.029	0.003	0.157	0.093	1.251
cg20735041	MUSTN1	0.812	0.087	0.755	0.009	3.264	27.181	0.003	0.157	-0.057	0.730
cg08564027		0.913	0.082	0.808	0.047	3.972	9.416	0.003	0.157	-0.105	1.399
cg11187245	HLA-B	0.599	0.067	0.540	0.025	3.436	17.582	0.003	0.160	-0.059	0.971
cg07684647		0.679	0.351	0.904	0.006	-3.272	25.065	0.003	0.163	0.225	0.715
cg27041096	COLEC10	0.667	0.071	0.597	0.031	3.571	13.838	0.003	0.163	-0.070	1.085
cg11265839	ELK3	0.707	0.075	0.788	0.036	-3.690	11.927	0.003	0.163	0.081	1.180
cg25106783	PRR4;TAS2R43;PRH1	0.716	0.077	0.779	0.025	-3.348	20.356	0.003	0.163	0.063	0.908
cg11752699	HLA-DRB6	0.414	0.260	0.233	0.053	3.219	28.625	0.003	0.164	-0.181	0.773
cg16514085	HLA-DRB1	0.384	0.352	0.668	0.399	-4.060	8.519	0.003	0.164	0.436	0.819
cg21457401	PNLDC1	0.444	0.065	0.391	0.021	3.321	21.307	0.003	0.164	-0.053	0.889
cg00236261	CCDC3	0.883	0.103	0.954	0.021	-3.208	28.722	0.003	0.165	0.071	0.767
cg21698879	HLA-DRB5	0.830	0.112	0.919	0.035	-3.308	21.747	0.003	0.165	0.089	0.880
cg06679334		0.449	0.071	0.381	0.030	3.502	14.792	0.003	0.165	-0.068	1.042
cg04566512		0.829	0.064	0.747	0.037	3.924	9.316	0.003	0.165	-0.082	1.389
cg23318020		0.384	0.109	0.302	0.030	3.256	24.491	0.003	0.165	-0.082	0.836
cg17416722	HLA-DRB1	0.201	0.230	0.055	0.008	3.238	25.290	0.003	0.166	-0.147	0.709
cg00440797	HLA-DRB5	0.247	0.225	0.099	0.030	3.202	28.362	0.003	0.166	-0.148	0.731
cg27639721	ATHL1	0.269	0.070	0.196	0.033	3.618	12.466	0.003	0.166	-0.073	1.139
cg11620135	BAIAP2	0.825	0.176	0.937	0.010	-3.228	25.790	0.003	0.167	0.112	0.711
cg24506197	PTPRN2	0.379	0.066	0.435	0.023	-3.333	19.492	0.003	0.167	0.055	0.915
cg07668501		0.228	0.080	0.134	0.043	3.796	10.165	0.003	0.167	-0.095	1.293
cg23352245		0.903	0.127	0.984	0.005	-3.228	25.346	0.003	0.168	0.081	0.707
cg23170091	MUC4	0.681	0.083	0.758	0.034	-3.446	15.521	0.003	0.168	0.077	1.010

cg10104420	HLA-DRB6	0.790	0.134	0.877	0.017	-3.189	28.084	0.003	0.169	0.087	0.724
cg16268937		0.683	0.340	0.898	0.008	-3.213	25.157	0.004	0.173	0.214	0.703
cg24947637		0.784	0.188	0.912	0.037	-3.170	28.817	0.004	0.173	0.128	0.755
cg03466717	ZNF423	0.643	0.075	0.696	0.018	-3.179	27.129	0.004	0.176	0.053	0.787
cg17597639	SDHAP3	0.134	0.072	0.089	0.004	3.190	25.819	0.004	0.177	-0.045	0.702
cg01682111	UNKL	0.403	0.078	0.332	0.031	3.390	15.694	0.004	0.182	-0.070	0.990
cg11821245	LDHC	0.621	0.236	0.770	0.019	-3.164	26.491	0.004	0.183	0.149	0.702
cg07414525	CHL1	0.628	0.067	0.550	0.037	3.735	10.027	0.004	0.183	-0.078	1.279
cg00935504	HAGH	0.601	0.066	0.737	0.060	-4.541	6.033	0.004	0.183	0.136	2.143
cg26655517		0.355	0.097	0.254	0.047	3.578	11.776	0.004	0.183	-0.102	1.149
cg10947831		0.319	0.213	0.502	0.080	-3.317	17.177	0.004	0.188	0.183	0.944
cg17201438	UBE2O	0.680	0.077	0.628	0.015	3.122	28.694	0.004	0.190	-0.052	0.747
cg13896699	DNAH5	0.670	0.155	0.794	0.053	-3.243	19.789	0.004	0.191	0.125	0.886
cg23432430		0.786	0.245	0.939	0.016	-3.136	26.014	0.004	0.195	0.153	0.692
cg06379361		0.098	0.128	0.018	0.007	3.133	25.815	0.004	0.197	-0.080	0.690
cg24932660	CD164L2	0.492	0.057	0.535	0.017	-3.165	23.179	0.004	0.197	0.043	0.827
cg06307913	PRDM8	0.483	0.080	0.389	0.045	3.690	9.809	0.004	0.197	-0.094	1.276
cg01567173		0.909	0.066	0.967	0.026	-3.319	16.051	0.004	0.197	0.058	0.963
cg02527811	C2orf54	0.748	0.176	0.857	0.016	-3.111	26.853	0.004	0.198	0.110	0.693
cg03030267	GNASAS;MIR298;MIR296	0.461	0.098	0.400	0.010	3.102	27.086	0.004	0.198	-0.061	0.693
cg25170017	EHD1	0.325	0.086	0.262	0.024	3.134	24.501	0.004	0.198	-0.063	0.805
cg04523090		0.676	0.083	0.590	0.041	3.515	11.633	0.004	0.198	-0.086	1.134
cg06373870	PRDM8	0.542	0.075	0.439	0.049	3.884	8.178	0.004	0.198	-0.103	1.470
cg11438287	COL4A2	0.779	0.179	0.213	0.242	4.981	4.882	0.004	0.198	-0.566	3.096
