# Supplementary material for: Differential genome-wide DNA methylation patterns in childhood obesity
Source: BMC Res Notes. 2019 Mar 25;12:174. doi: 10.1186/s13104-019-4189-0 (PMC6434834; doi:10.1186/s13104-019-4189-0)
Supplement: Supplementary file 2 — Additional file 2: Table S2. 52 canonical pathways classified with IPA. IPA classified and revealed 52 canonical pathways based on the 143 genes differentially methylated between obese and non-obese groups. The biological functions of these pathways were predominantly involved in immune system. [file 13104_2019_4189_MOESM2_ESM.pdf]

Table S2: 52 canonical pathways classified with IPA

	Ingenuity Canonical Pathways	-log(p-value)	Ratio	Genes
1	Antigen Presentation Pathway	7.5E00	1.35E-01	NLRC5,HLA-DRB1,HLA-B,TAP1,HLA-DRB5
2	Graft-versus-Host Disease Signaling	3.64E00	6.25E-02	HLA-DRB1,HLA-B,HLA-DRB5
3	Autoimmune Thyroid Disease Signaling	3.61E00	6.12E-02	HLA-DRB1,HLA-B,HLA-DRB5
4	Cdc42 Signaling	3.1E00	2.4E-02	HLA-DRB1,HLA-B,IQGAP1,HLA-DRB5
5	Allograft Rejection Signaling	2.89E00	3.49E-02	HLA-DRB1,HLA-B,HLA-DRB5
6	Crosstalk between Dendritic Cells and Natural Killer Cells	2.85E00	3.37E-02	HLA-DRB1,HLA-B,HLA-DRB5
7	OX40 Signaling Pathway	2.85E00	3.37E-02	HLA-DRB1,HLA-B,HLA-DRB5
8	Communication between Innate and Adaptive Immune Cells	2.82E00	3.3E-02	HLA-DRB1,HLA-B,HLA-DRB5
9	Type I Diabetes Mellitus Signaling	2.59E00	2.73E-02	HLA-DRB1,HLA-B,HLA-DRB5
10	B Cell Development	2.49E00	5.88E-02	HLA-DRB1,HLA-DRB5
11	Nur77 Signaling in T Lymphocytes	2.05E00	3.51E-02	HLA-DRB1,HLA-DRB5
12	Catecholamine Biosynthesis	2E00	2.5E-01	DBH
13	Biotin-carboxyl Carrier Protein Assembly	2E00	2.5E-01	HLCS
14	Dendritic Cell Maturation	2E00	1.68E-02	HLA-DRB1,HLA-B,HLA-DRB5
15	Calcium-induced T Lymphocyte Apoptosis	1.96E00	3.12E-02	HLA-DRB1,HLA-DRB5
16	CMP-N-acetylneuraminate Biosynthesis I (Eukaryotes)	1.91E00	2E-01	GNE
17	Hepatic Fibrosis / Hepatic Stellate Cell Activation	1.89E00	1.52E-02	COL4A1,COL4A2,COL9A2
18	T Helper Cell Differentiation	1.87E00	2.82E-02	HLA-DRB1,HLA-DRB5
19	IL-4 Signaling	1.81E00	2.63E-02	HLA-DRB1,HLA-DRB5
20	Altered T Cell and B Cell Signaling in Rheumatoid Arthritis	1.69E00	2.27E-02	HLA-DRB1,HLA-DRB5
21	iCOS-iCOSL Signaling in T Helper Cells	1.53E00	1.85E-02	HLA-DRB1,HLA-DRB5
22	CD28 Signaling in T Helper Cells	1.46E00	1.69E-02	HLA-DRB1,HLA-DRB5
23	PKCθ Signaling in T Lymphocytes	1.46E00	1.69E-02	HLA-DRB1,HLA-DRB5
24	Role of NFAT in Regulation of the Immune Response	1.17E00	1.17E-02	HLA-DRB1,HLA-DRB5
25	Glutathione-mediated Detoxification	1.14E00	3.33E-02	GSTM5
26	NRF2-mediated Oxidative Stress Response	1.13E00	1.11E-02	GSTM5,ENC1
27	Cytotoxic T Lymphocyte-mediated Apoptosis of Target Cells	1.12E00	3.12E-02	HLA-B
28	Oncostatin M Signaling	1.09E00	2.94E-02	MT2A
29	Interferon Signaling	1.09E00	2.94E-02	TAP1
30	Notch Signaling	1.04E00	2.63E-02	DTX1
31	Neuroprotective Role of THOP1 in Alzheimer's Disease	1.02E00	2.5E-02	HLA-B
32	Primary Immunodeficiency Signaling	9.15E-01	1.92E-02	TAP1
33	Protein Ubiquitination Pathway	8.78E-01	7.84E-03	HLA-B,TAP1
34	Pyridoxal 5'-phosphate Salvage Pathway	8.31E-01	1.56E-02	PDXK
35	Remodeling of Epithelial Adherens Junctions	8.07E-01	1.47E-02	IQGAP1
36	PEDF Signaling	7.89E-01	1.41E-02	ARHGAP22
37	Caveolar-mediated Endocytosis Signaling	7.84E-01	1.39E-02	HLA-B
38	TREM1 Signaling	7.68E-01	1.33E-02	NLRC5
39	CTLA4 Signaling in Cytotoxic T Lymphocytes	7.05E-01	1.14E-02	HLA-B
40	Virus Entry via Endocytic Pathways	7.01E-01	1.12E-02	HLA-B
41	Rac Signaling	6.41E-01	9.62E-03	IQGAP1
42	Gαs Signaling	6.23E-01	9.17E-03	HCAR3
43	Aryl Hydrocarbon Receptor Signaling	5.3E-01	7.14E-03	GSTM5
44	Epithelial Adherens Junction Signaling	5.15E-01	6.85E-03	IQGAP1
45	Germ Cell-Sertoli Cell Junction Signaling	4.82E-01	6.25E-03	IQGAP1
46	IL-8 Signaling	4.35E-01	5.46E-03	IQGAP1
47	Actin Cytoskeleton Signaling	3.77E-01	4.61E-03	IQGAP1
48	LPS/IL-1 Mediated Inhibition of RXR Function	3.74E-01	4.57E-03	GSTM5
49	cAMP-mediated signaling	3.74E-01	4.57E-03	HCAR3
50	Systemic Lupus Erythematosus Signaling	3.73E-01	4.55E-03	HLA-B
51	Signaling by Rho Family GTPases	3.53E-01	4.27E-03	IQGAP1
52	G-Protein Coupled Receptor Signaling	3.25E-01	3.91E-03	HCAR3
53	Xenobiotic Metabolism Signaling	3.07E-01	3.69E-03	GSTM5
